# Dually functioned core-shell NaYF_4_:Er^3+^/Yb^3+^@NaYF_4_:Tm^3+^/Yb^3+^ nanoparticles as nano-calorifiers and nano-thermometers for advanced photothermal therapy

**DOI:** 10.1038/s41598-017-11897-4

**Published:** 2017-09-19

**Authors:** Yanqiu Zhang, Baojiu Chen, Sai Xu, Xiangping Li, Jinsu Zhang, Jiashi Sun, Hui Zheng, Lili Tong, Guozhu Sui, Hua Zhong, Haiping Xia, Ruinian Hua

**Affiliations:** 1 Department of Physics, Dalian Maritime University, Dalian, 116026 People’s Republic of China; 2 Key laboratory of Photo-electronic Materials, Ningbo University, Ningbo, 315211 People’s Republic of China; 3 College of Life Science, Dalian Nationalities University, Dalian, 116600 People’s Republic of China

## Abstract

To realize photothermal therapy (PTT) of cancer/tumor both the photothermal conversion and temperature detection are required. Usually, the temperature detection in PTT needs complicated instruments, and the therapy process is out of temperature control in the present investigations. In this work, we attempt to develop a novel material for achieving both the photothermal conversion and temperature sensing and control at the same time. To this end, a core-shell structure with NaYF_4_:Er^3+^/Yb^3+^ core for temperature detection and NaYF_4_:Tm^3+^/Yb^3+^ shell for photothermal conversion was designed and prepared. The crystal structure and morphology of the samples were characterized by X-ray diffraction (XRD), scanning electron microscopy (SEM) and transmission electron microscopy (TEM). Furthermore, the temperature sensing properties for the NaYF_4_:Er^3+^/Yb^3+^ and core-shell NaYF_4_:Er^3+^/Yb^3+^@NaYF_4_:Tm^3+^/Yb^3+^ nanoparticles were studied. It was found that the temperature sensing performance of the core-shell nanoparticles did not become worse due to coating of NaYF_4_:Tm^3+^/Yb^3+^ shell. The photothermal conversion behaviors were examined in cyclohexane solution based on the temperature response, the NaYF_4_:Er^3+^/Yb^3+^@NaYF_4_:Tm^3+^/Yb^3+^ core-shell nanoparticles exhibited more effective photothermal conversion than that of NaYF_4_:Er^3+^/Yb^3+^ nanoparticles, and a net temperature increment of about 7 °C was achieved by using the core-shell nanoparticles.

## Introduction

In the clinical research and practice, hyperthermia alone or its combination with other therapies including surgery, radiation therapy, chemotherapy and anticancer drug, have been applied in the control and treatment for tumors and cancers^1–5^. Due to the fact that the tumor cells and tissues are sensitive to the temperature above normal^6^, thus hyperthermia works based on increasing the temperature of lesion location for a period of time and killing cancerous cells with less damage on normal cells and tissues. The successful cases of treatments on breast, head and neck tumors have indicated that hyperthermia as adjuvant therapy effectively controls tumors and reduces recurrence^7^.

As an interesting alternative for hyperthermia, PTT has attracted much attention due to its advantages of less side effects, minimal invasiveness, high specificity and short treatment period^8–11^. The mechanism for PTT is based on the photothermal conversion agents that convert their absorbed light energy into heat to thermally ablate targeted cancer cells at a certain temperature^8^. Thus, external excitation light sources, temperature measurements and photothermal conversion materials are imperative conditions for the whole process of PTT. Firstly, for the excitation source, the light lying in the biological windows (first window: 700–980 nm, second window: 1000–1400 nm) is commonly used as excitation source^12,13^. In fact, though the excitation wavelength is located at the biological windows, the penetration depth of the light is finite. However, the tumors located at superficial tissues such as galactophore cancer and cutaneum carcinoma ask for short penetration distance of excitation light, and the tumors in deep tissues can also be treated in assistance of medical endoscope technique^14^. When exposed in the spectral windows, the photothermal agents can selectively work in the lesions where the agents are incorporated into, and extremely avoid unnecessary injury to the surrounding normal tissues. This is to say, the tumors, which are targeted by the photothermal agents, can reach the expected temperature and be cured before the healthy issue is injured. Moreover, the excitation lights, whose wavelengths fall into the biological windows, can penetrate biotic tissues with a few centimeters depth, which makes deep-tissue hyperthermia treatments possible. Secondly, for the temperature sensing, precisely measuring the temperature is crucial to control heat release and treatment time maintained in the location of tumor cells. Currently, a variety of temperature-measuring methods, such as infrared thermal imaging^15,16^, magnetic resonance imaging^17^, photoacoustic technique^18,19^ and ultrasound^20^, have been extensively used. The non-contact thermometry still suffers from many applied limitations, although these methods can be used to detect the average temperature or surface temperature of the photothermal heaters at different heating time, excitation power and thermal dosage. For instance, real-time thermal measurement of tumors located in surface can be achieved by infrared thermal imaging, but the temperature in deep tissue fails to be detected. Photoacoustic measurement gives high resolution images, but the sensitivity is lower. The resolution and sensitivity of magnetic resonance imaging measurement are higher, whereas the temperature feedback time is long and the equipment is heavy and costly. Considering these disadvantages of temperature monitoring at present, it is vitally important to obtain real-time thermal feedback of photothermal heaters in deep tumors for controlling temperature and thermal dosage injection in PTT process. Lastly, the photothermal conversion materials are placed in lesions to enhance the light absorption and heat conversion in the tumors. The rapid development of biotechnology and nanotechnology provides several kinds of photothermal conversion materials including metallic nanoparticles^21,22^, magnetic nanoparticles^23^, semiconductor quantum dots (CdSe, CdTe)^24,25^, carbon-based nanomaterials (graphene, carbon nanotubes)^26,27^, organic nanoparticles (dyes, polymer, biomacromolecule)^28–30^ for PTT. Despite the effective conversion from light energy to heat, these photothermal conversion materials still exhibit some limitations such as weak biological compatibility and long-term toxicity in clinical applications^31^. The body may suffer from the acute inflammation and cell apoptosis which are caused by poor-metabolized metallic nanoparticles^32,33^. Carbon nanotubes not only suppress the immune system but also cause pulmonary inflammation and granuloma after bronchial instillation^34,35^. Graphene produces serious oxidative stress and irritates the skin, eye and respiratory system after intravenous injection^26,36^. Therefore, novel photothermal agents with low systemic toxicity and high conversion efficiency require to be developed for meeting clinical demands.

Rare earth (RE) doped upconversion nanoparticles (UCNPs) with many advantages of unique anti-Stokes fluorescence, large penetration depth of infrared (IR) excitation light into tissues, low background noise, high chemical stability and low toxicity, have been extensively investigated in biodetection, drug delivery, fluorescence labeling and imaging and optical temperature sensors^37–42^. Typically, Yb^3+^ is widely used as photo-sensitizer due to its larger absorption cross section at 980 nm, and then it could transfer the absorbed energy to the quenchers (for example Sm^3+^) which can accept the energy and produce heat via nonradiative relaxation^43^. Under 808 nm excitation, Nd^3+^ is commonly selected as sensitizer to absorb the light energy effectively. The photothermal conversion effect induced by 808 nm laser irradation in Sm^3+^/Nd^3+^ co-doped NaY(WO_4_)_2_ microstructures was investigated^44^. The advantage of adopting Nd^3+^ as both the photothermal conversion and temperature sensing is that both the excitation emission wavelengths are in the biological window and the deep tissue treatment can be achieved^45,46^. These investigations revealed that the RE doped nanoparticles can be photothermal conversion candidates, and can also achieve effective temperature detection at the exact position where the RE doped nanoparticles located at. Compared with the traditional temperature measurements, the kind of non-contact optical temperature sensor can monitor and feed back the real-time temperature of tumors in deep tissues. From what mentioned above, combining the photothermal conversion and temperature sensing in one nanoplatform would be beneficial to the practical application in advanced PTT^47^. Although scientists have made significant progresses in the vivo photothermal therapy treatments by using optical temperature sensing nanoparticles to read temperature^48,49^, developing novel nanoparticles possessing both the photothermal conversion and temperature sensing is still a challenge^50–52^.

According to the above analysis, in this work, we intend to develop a nano-system in which the functions of photothermal conversion and temperature sensing are expected to be integrated. Thereby, a core-shell nanostructure composed of the NaYF_4_:Er^3+^/Yb^3+^ core and NaYF_4_:Tm^3+^/Yb^3+^ shell is designed. The reason of choosing this nanostructure is that Er^3+^/Yb^3+^ couple can realize temperature sensing^53–56^, and Tm^3+^/Yb^3+^ couple exhibited strong photo-heat conversion^57^, meanwhile the NaYF_4_ core-shell structured nanoparticles can be easily prepared via thermal decomposition route. Therefore, in this work, the NaYF_4_:Er^3+^/Yb^3+^@NaYF_4_:Tm^3+^/Yb^3+^ core-shell nanoparticles were prepared, and their crystal structure and morphology were characterized by means of XRD, SEM and TEM. Furthermore, the spectroscopic study indicated that the core-shell nanoparticles presented excellent temperature sensing and photothermal conversion performance. It should be emphasized that the heat converted from the core-shell structure comes not only from the NaYF_4_:Tm^3+^/Yb^3+^ shell but also from the NaYF_4_:Er^3+^/Yb^3+^ core, and the former contributes more heat than that of the latter. This is because of the fact that the full upconversion luminescence process of the NaYF_4_:Er^3+^/Yb^3+^ core is accompanied by the nonradiative transitions from all the populated energy levels, and these nonradiative transitions will produce heat.

## Results and Discussion

### Structure and morphology of NaYF_4_ core and core-shell structure

**Figure 1:**
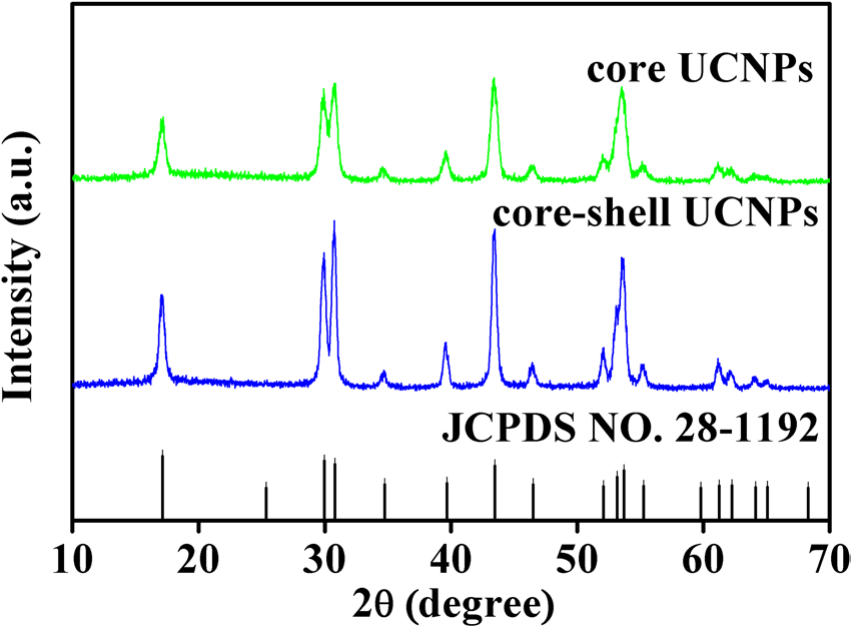
XRD patterns of the NaYF_4_:Er^3+^/Yb^3+^ core and NaYF_4_: Er^3+^/Yb^3+^@NaYF_4_: Tm^3+^/Yb^3+^ core-shell UCNPs, the standard card of β-NaYF_4_ (JCPDS: no. 28-1192) is shown as reference.

The XRD patterns for the pulverized NaYF_4_: Er^3+^/Yb^3+^ core and NaYF_4_: Er^3+^/Yb^3+^@NaYF_4_: Tm^3+^/Yb^3+^ core-shell are shown in Fig. 1. All the diffraction peaks can be well indexed in accordance with diffraction pattern of hexagonal NaYF_4_ powder (in JCPDS card no. 28-1192), which indicates that the samples prepared are free of impurities, neither the introduction of RE^3+^ into the host nor the shell coating has effect on the crystal phase. The diffraction peaks of NaYF_4_:Er^3+^/Yb^3+^@NaYF_4_:Tm^3+^/Yb^3+^ core-shell nanoparticles are slightly narrower than that of the core. From the diffraction peaks at 2*θ* angle of 17°, the crystallite sizes of the samples are estimated based on the Debye-Scherrer formula ($D=\frac{K\lambda }{BCOS\theta }$) to be approximately 21.2 nm for the core and 26.2 nm for the core-shell nanoparticles, respectively. This means that the expected NaYF_4_:Er^3+^/Yb^3+^@NaYF_4_:Tm^3+^/Yb^3+^ core-shell structure might be received via the synthesis approach stated in Experimental section.

**Figure 2:**
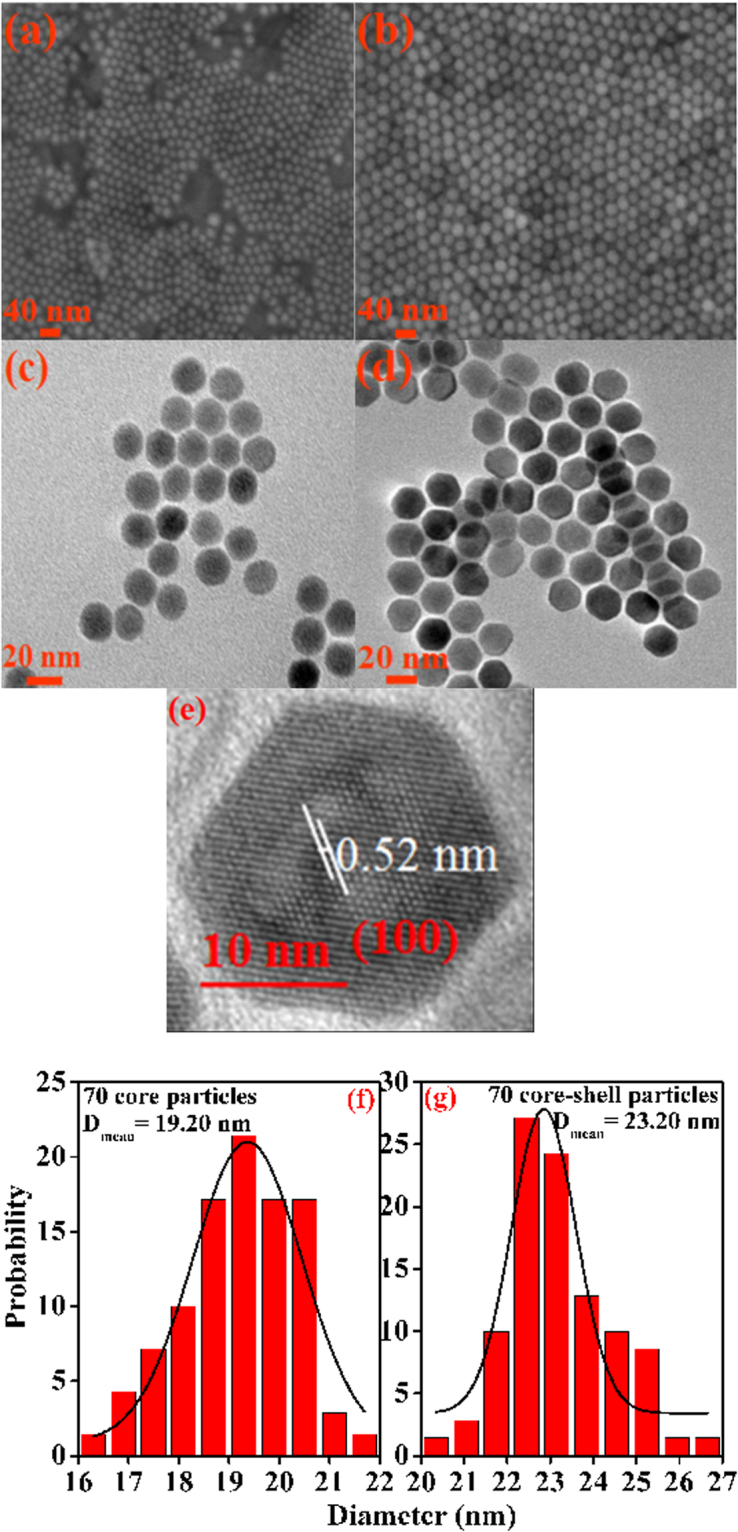
(**a**) and (**b**) SEM images of core and core-shell UCNPs, (**c**) and (**d**) TEM images of core and core-shell UCNPs, (**e**) HRTEM images of the core-shell sample, (**f**) and (**g**) Size distribution histograms of the core and core-shell nanoparticles derived from the TEM images.

To observe the microscopic morphology for the NaYF_4_:Er^3+^/Yb^3+^ core and NaYF_4_:Er^3+^/Yb^3+^@NaYF_4_:Tm^3+^/Yb^3+^ core-shell structure, SEM images were taken and are shown in Fig. 2a,b, respectively. It can be seen from the images that both core and core-shell samples are composed of uniform and regular sphere-like particles. For more clearly observing histological morphology of the samples, the TEM images of the synthesized core and core-shell samples were measured and are displayed in Fig. 2c,d, respectively. It is found that particles in the naked core sample in Fig. 2c are monodispersed and sphere-shaped particles, but fewer particles showed hexagonal shape. Figure 2d for the core-shell sample displays that the particles remain monodispersed, but the morphology of all particles is of hexagonal shape. To estimate the particle sizes of the core and core-shell samples, the sizes of 70 particles for each sample were measured from the TEM images by using noncommercial software. The particle size histograms of core and core-shell samples are shown in Fig. 2f,g, respectively. It is found from Fig. 2f,g that the particle size distribution is narrow, and the average diameter of core sample is determined to be 19.2 nm, and the mean particle size of core-shell sample increases up to 23.2 nm. The results are nearly consistent with the calculation results derived from XRD patterns. These statistical results for particle sizes imply that NaYF_4_:Er^3+^/Yb^3+^ nanoparticles were successfully coated with about 2.0 nm thickness NaYF_4_:Tm^3+^/Yb^3+^ shell. Figure 2e exhibits the HRTEM images of core-shell UCNPs, the two adjacent lattice fringes with a lattice spacing of 0.52 nm matches well with that of the (100) plane in hexagonal NaYF_4_. The obvious lattice fringes in the HRTEM images further confirm the core-shell structure possesses high crystallinity.

### Temperature sensing properties of NaYF_4_:Er^3+^/Yb^3+^ and NaYF_4_:Er^3+^/Yb^3+^@NaYF_4_: Tm^3+^/Yb^3+^ core-shell particles

Usually, the temperature at tissue surface is relatively easy to be detected with non-contact thermal camera, but the PTT process of tumor located in deep tissue would require real time temperature reading. Therefore, it is necessary to develop a non-contact thermometer. The present studied NaYF_4_:Er^3+^/Yb^3+^@NaYF_4_:Tm^3+^/Yb^3+^ core-shell in which Er^3+^ has two adjacent levels, namely ^4^S_3/2_ and ^2^H_11/2_ in thermal equilibrium, may be a good temperature sensing material. Because the emission intensity ratio of ^2^H_11/2_ → ^4^I_15/2_ to ^4^S_3/2_ → ^4^I_15/2_ is equal to the population ratio of ^2^H_11/2_ to ^4^S_3/2_ which is only dependent on the sample temperature for a certain system. These relations can be mathematically expressed as below.

1 $${\boldsymbol{R}}{\boldsymbol{=}}\frac{{{\boldsymbol{I}}}_{{\boldsymbol{H}}}}{{{\boldsymbol{I}}}_{{\boldsymbol{S}}}}{\boldsymbol{=}}{\boldsymbol{C}}{\bf{\exp }}{\boldsymbol{(}}{\boldsymbol{-}}{\boldsymbol{\Delta }}{\boldsymbol{E}}{\boldsymbol{/}}{\boldsymbol{kT}}{\boldsymbol{)}}$$

In above equation, ***R*** stands for the fluorescence intensity ratio; ***I***_***H***_ and ***I***_***S***_ represents the integrated emission intensities of ^2^H_11/2_ → ^4^I_15/2_ and ^4^S_3/2_ → ^4^I_15/2_, respectively; ***C*** is a constant depending on the doped RE^3+^ and host materials; ***ΔE*** is the energy gap between ^2^H_11/2_ and ^4^S_3/2_ states, ***k*** is the Boltzmann constant, and ***T*** is the absolute temperature. Therefore, the sample temperature can be readily derived by taking the measured fluorescence intensity ratio into equation (1) as long as ***ΔE*** and ***C*** are confirmed.

**Figure 3:**
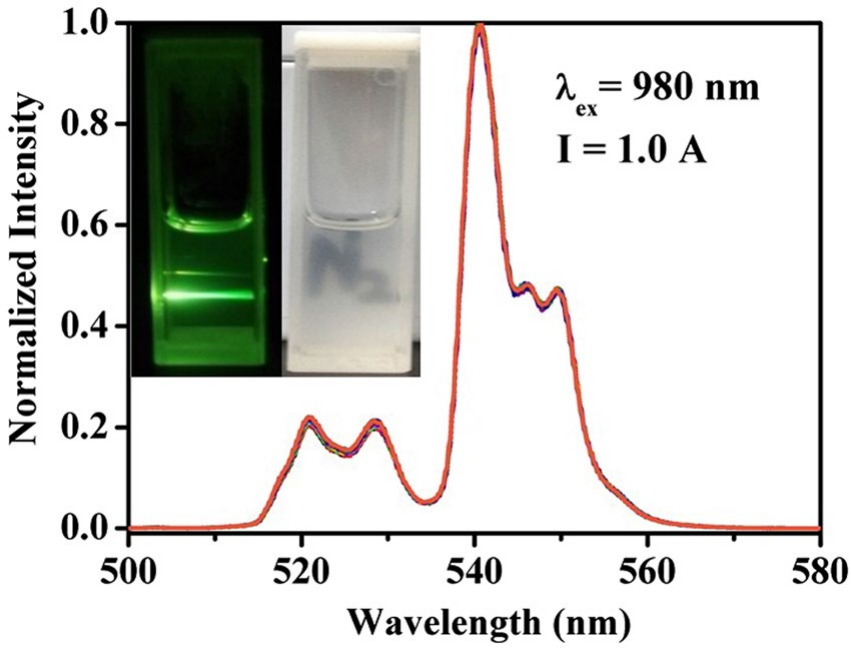
Normalized green UC emission spectra of NaYF_4_:Er^3+^/Yb^3+^@NaYF_4_:Tm^3+^/Yb^3+^ core-shell sample measured at different moments within 70 min when continuously excited by 980 nm fiber laser working at current of 1 A. The inset shows digital photos of core-shell particles in cyclohexane solution under daylight (right side) and under 980 nm irradiation in dark background (left side).

In order to obtain the parameters ***C*** and ***ΔE*** for the NaYF_4_:Er^3+^/Yb^3+^ and NaYF_4_:Er^3+^/Yb^3+^@NaYF_4_:Tm^3+^/Yb^3+^ core-shell particles, the temperature calibration experiments were carried out. In doing so, the cyclohexane solution containing OA-capped NaYF_4_:Er^3+^/Yb^3+^ particles or OA-capped NaYF_4_:Er^3+^/Yb^3+^@NaYF_4_:Tm^3+^/Yb^3+^ core-shell particles were prepared. The reason for using cyclohexane is that the OA-capped UCNPs could be well dispersed in cyclohexane to form stable colloidal solution directly without further surface modification, and the colloidal solution can maintain transparent and homogeneous for several days. The inset of Fig. 3 shows the images of the solution under irradiation of daylight and 980 nm fiber laser in dark background. The solution in both cases is homogeneous and ready for the spectral measurements. In the course of temperature calibration, 2 mL solution (4 Wt% of the nanoparticles) in cuvette was firstly heated via water bath to 373 K, and then moved to the sample chamber of the spectrometer for spectral measurement. The UC emission spectra at various temperatures were recorded when the solution was naturally cooled down until room temperature, and the solution temperatures were recoded via a K-type thermocouple connected to a proportional-integral-differential (PID) controller. It should be pointed out that when studying the temperature sensing property, the excitation power should be set as low as possible in order to avoid the laser-induced thermal effect^39,40,43,57^. To check this effect, the green UC emission spectra for the core-shell solution at room temperature were measured at different time under 980 nm fiber laser working at electric current of 1.0 A, and are shown in Fig. 3. It can be seen that all the spectral lines are accurately overlapped, thus implying that under the laser irradiation the solution temperature does not change obviously with time, that is to say, the thermal effect induced by constant laser irradiation can be omitted under these conditions. Therefore, these conditions except for the solution temperature were kept for the full processes of spectral measurements. It should be mentioned that the excitation power densities in this work were about 0.24 × 10^2^ W/cm^2^ and 1.29 × 10^2^ W/cm^2^ when the laser working currents were 1.0 and 2.0 A.

**Figure 4:**
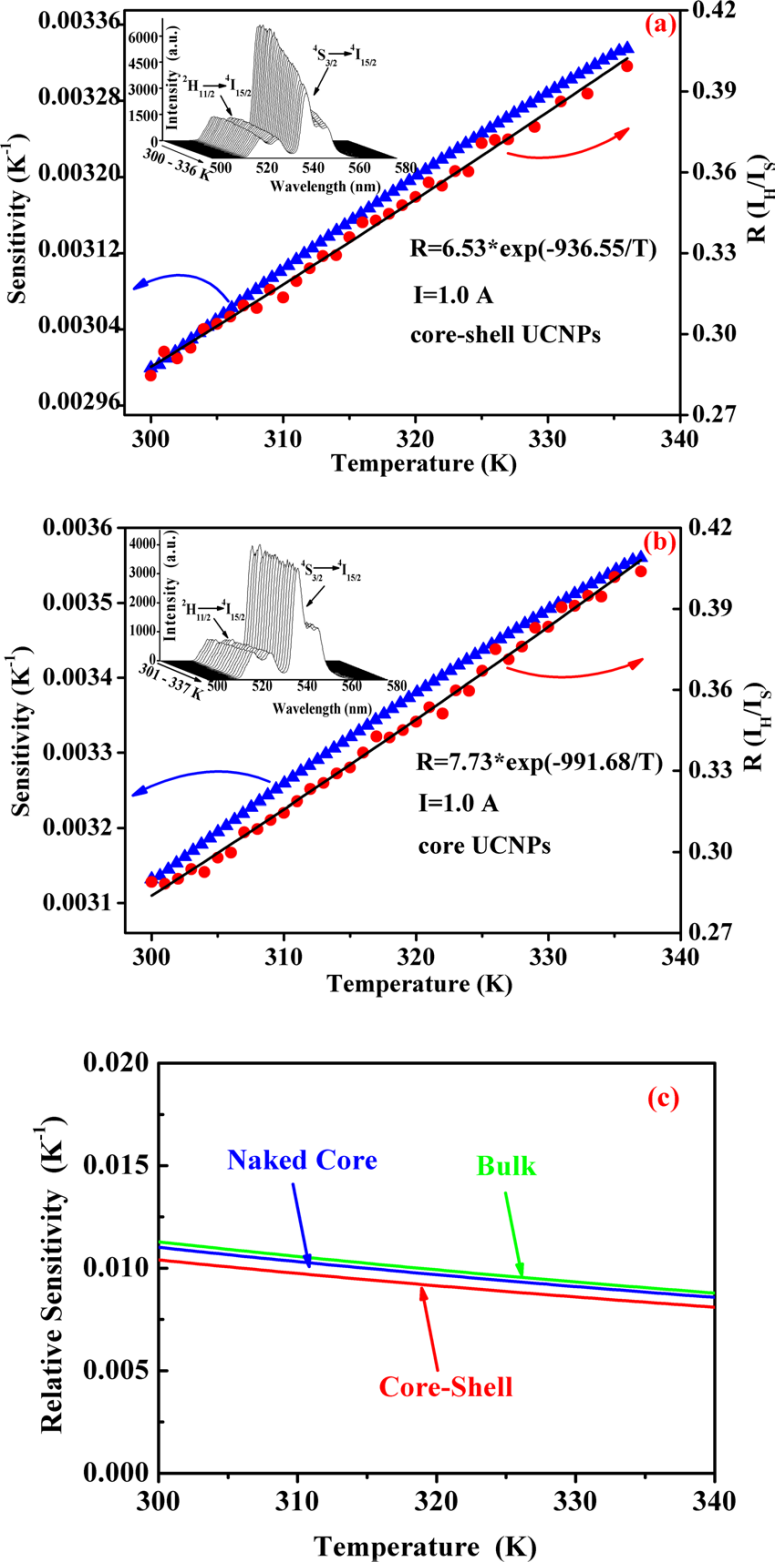
Dependence of green emission intensity ratio (I_R_/I_S_) (•) and sensitivity (▴) on sample temperature, the solid curves represent the fitting curves of experimental data to equation (1) (**a**) for NaYF_4_:Er^3+^/Yb^3+^@ NaYF_4_:Tm^3+^/Yb^3+^ core-shell particles and (**b**) for NaYF_4_:Er^3+^/Yb^3+^ particles. The inserts show the upconversion spectra for both the samples measured at different temperatures. (**c**) Shows the dependence of relative sensitivities for the naked core, core-shell structure and the bulk on the temperature.

The insert in Fig. 4a shows the UC spectra for solution with core-shell sample at various temperatures ranged from 300 to 336 K. It can be seen that the emission intensities of ^4^S_3/2_ → ^4^I_15/2_ and ^2^H_11/2_ → ^4^I_15/2_ both decrease with increasing solution temperature, which is caused by the higher nonradiative transition rate of ^4^S_3/2_ level^58^. The integrated intensities at different temperatures for ^4^S_3/2_ → ^4^I_15/2_ and ^2^H_11/2_ → ^4^I_15/2_ transitions were calculated, and then the fluorescence intensity ratios were derived. The solid circles in Fig. 4a represent the dependence of integrated fluorescence intensity ratio on the solution temperature, and the solid line shows the fitting curve by using equation (1). The free parameters ***C*** and ***ΔE***/***k*** were confirmed to be 6.53 and 936.55 K from the fitting processes. In view of the above, the temperature response curve for the core-shell nanoparticles can be received by taking the parameters ***C*** and ***ΔE***/***k*** into equation (1). When the temperature response curve is known, the sensitivity (***S***) for temperature detection can be defined as derivative of fluorescence intensity ratio (***R***) with respect to temperature (***T***), and written as follows,

2 $${\boldsymbol{S}}=\frac{{\boldsymbol{dR}}}{{\boldsymbol{dT}}}={\boldsymbol{C}}{\bf{\exp }}(-{\boldsymbol{\Delta }}{\boldsymbol{E}}/{\boldsymbol{kT}})({\boldsymbol{\Delta }}{\boldsymbol{E}}/{\boldsymbol{k}}{{\boldsymbol{T}}}^{{\bf{2}}})$$

On the basis of equation (2), the temperature sensing sensitivity curve (solid triangle dots) was obtained and is shown in Fig. 4a. It is confirmed that the sensitivity ranges from 0.0030 to 0.0033 K^−1^ in the temperature range of 300–336 K.

It should be mentioned that in above-studied core-shell structure the temperature sensing component, namely the NaYF_4_:Er^3+^/Yb^3+^ core, is covered by NaYF_4_:Tm^3+^/Yb^3+^ shell which is expected to play the part of calorifiers. Therefore, it is necessary to clarify if the temperature sensing performance of the core-shell particles becomes worse. For this purpose, the temperature sensing properties for the NaYF_4_:Er^3+^/Yb^3+^ particles were studied in a analogical way as done for the core-shell sample. The obtained results are shown in Fig. 4b where the insert depicts the UC spectra measured at various temperatures, and the solid circles present the dependence of fluorescence intensity ratio on the sample temperature, the solid up-triangles gives the temperature-dependent sensitivity. The parameters ***C*** and ***ΔE***/***k*** in equation (1) were confirmed to be 7.73 and 991.68 K, and the sensitivity increases from 0.003 to 0.0035 K^−1^ as temperature increases from 301 to 337 K. In comparison, the temperature sensing properties of the naked core and the core-shell are almost the same, thus indicating that the temperature sensing performance of NaYF_4_:Er^3+^/Yb^3+^ component in core-shell structure does not abate after coating with NaYF_4_:Tm^3+^/Yb^3+^ shell. It should be mentioned that the UC luminescence intensity of NaYF_4_:Er^3+^/Yb^3+^@ NaYF_4_:Tm^3+^/Yb^3+^ core-shell is 1.6 times higher than that of the naked NaYF_4_:Er^3+^/Yb^3+^ core under the same experimental conditions. The mechanism of luminescence enhancement in the core-shell structure can not be easily deduced from the present spectral data, nonetheless, the intense UC emissions would be beneficial to the temperature measurement in the PTT application, since intense enough fluorescence signal would lower the requirement for high performance sensor in the spectral measurements, and meanwhile make the extraction of UC emissions from tissue easier. Here, we should emphasize that both the green emissions of Er^3+^ are in the outside of the biological windows, therefore the core-shell nanoparticles can not be used for the tumor treatment in deep tissue, but they may be useful for treatment of superficial tissue.

In fact, the temperature sensing of lanthanide ions has been widely studied^45,59–61^. To further evaluate the temperature sensing performance of the designed core-shell structure, the relative sensitivities for the naked core, core-shell structure and the bulk NaYF_4_:Er^3+^/Yb^3+^ were calculated in the temperature region from 300 to 340 K by using the data obtained in this work and our previous work^60^. The relative sensitivity is defined as ${S}_{R}=\frac{S}{R}$ where ***R*** and ***S*** are determined by Eqs (1) and (2). The calculated results are shown in Fig. 4c. From Fig. 4c it can be seen that all the relative sensitivities for these three particles decrease with increasing temperature, and the relative sensitivity for NaYF_4_:Er^3+^/Yb^3+^@ NaYF_4_:Tm^3+^/Yb^3+^ core-shell structure does not change obviously in comparison with the naked NaYF_4_:Er^3+^/Yb^3+^ cores and the bulk NaYF_4_:Er^3+^/Yb^3+^ produced by our group. To compare the relative sensitivity of our designed core-shell structure with those in references, the *ΔE*/*k* for NaYF_4_:Er^3+^/Yb^3+^ particles under different excitation wavelengths and in different forms including core-shell structures and different crystal phases are collected and listed in Table 1 ^62–71^. The 1^st^ column of Table 1 presents the size, morphology or structure; the phase and the excitation wavelength for each NaYF_4_:Er^3+^/Yb^3+^ particles are listed in 2^nd^ and 3^rd^ columns; the 4^th^ column contains the *ΔE*/*k* values for all the NaYF_4_:Er^3+^/Yb^3+^ samples; the last column gives references. From Table 1 it can be found that the parameter *ΔE*/*k* determining relative sensitivity of NaYF_4_:Er^3+^/Yb^3+^ particles changes greatly from sample to sample, and our samples exhibit moderate *ΔE*/*k* values amongst all the results from different research groups. In this study, the temperature sensing properties of the naked core and core-shell structure were derived under the condition that the nanoparticles were immersed in cyclohexane liquid. Here we should point out that the most recent investigation proves that the temperature sensing properties of rare earth doped nanoparticles depend also on the immersion liquid environment^72^, thus the obtained results can be different when they are used in *in-vivo* systems.

**Table 1:** ***Δ*** *E*/*k* for NaYF_4_:Er^3+^/Yb^3+^ with different sizes, morphologies and structures.

Status of NaYF_4_:Er^3+^/Yb^3+^	Crystal Phase	Excitation Length (nm)	***Δ*** *E*/*k* (k^−1^)	Refs.
core-shell NaYF_4_:Er^3+^/Yb^3+^@NaYF_4_:Nd^3+^/Yb^3+^	beta-	800	993.2±18.8	62
silane modified NaYF_4_:Er^3+^/Yb^3+^	beta-	980	1065.9	63
75 nm NaYF_4_:Er^3+^/Yb^3+^	alpha-	980	1025.8	60
rods, 200 nm in diameter, 400 nm in length, NaYF_4_:Er^3+^/Yb^3+^	beta-	980	1085.3	60
15 nm, NaGdF_4_:Er^3+^/Yb^3+^	beta-	980	1013±28	64
15 nm core, 5 nm shell NaGdF_4_:Er^3+^/Yb^3+^@NaYF_4_			1060±55	
15 nm core and 10 nm shell NaGdF_4_:Er^3+^/Yb^3+^@NaYF_4_			1035±44	
15 nm core and 15 nm shell NaGdF_4_:Er^3+^/Yb^3+^@NaYF_4_			1027±32	
27 nm, NaYF_4_:Er^3+^/Yb^3+^@SiO_2_	beta-	980	969	65
bulk NaYF_4_:Er^3+^/Yb^3+^/Li^+^	beta-	980	1198.2±41.18	66
polydimethylsiloxane stabled NaYF_4_:Er^3+^/Yb^3+^	beta-	980	1044.16	67
370 nm in diameter, 419 nm in length NaYF_4_:Er^3+^/Yb^3+^	beta-	980	748.9	68
nanosized NaYF_4_:Er^3+^/Yb^3+^	beta-	980	1028.5	69
nanosized NaYF_4_:Er^3+^/Yb^3+^@SiO_2_			1031.5	
bulk NaYF_4_:Er^3+^/Yb^3+^	beta-	980	1306.72±12.57	70
nanowire NaYF_4_:Er^3+^/Yb^3+^			1317.39±4.81	
nanorods NaYF_4_:Er^3+^/Yb^3+^			1343.14±29.35	
nanoplates NaYF_4_:Er^3+^/Yb^3+^			1397.41±9.39	
hexagonal microprisms 6 μm in length and 2μm in diameter NaYF_4_:Er^3+^/Yb^3+^	beta-	980	1082.1	71
19 nm, NaYF_4_:Er^3+^/Yb^3+^	beta-	980	936.55	This work
19 nm core, 4 nm shell NaYF_4_:Er^3+^/Yb^3+^@ NaYF_4_:Tm^3+^/Yb^3+^			991.68	

### Photothermal conversion effect of NaYF_4_ core-shell structure as nano-calorifier

In above section, the NaYF_4_:Er^3+^/Yb^3+^@NaYF_4_:Tm^3+^/Yb^3+^ core-shell particles were qualified for temperature sensing as good as NaYF_4_:Er^3+^/Yb^3+^ particles. The aim of this work is to develop bifunctional nanomaterials having both temperature sensing and photothermal conversion for PTT, therefore, in this section we will discuss about the photothermal conversion properties of the NaYF_4_:Er^3+^/Yb^3+^@NaYF_4_:Tm^3+^/Yb^3+^ core-shell particles.

To examine the photothermal conversion effect of the core-shell nanoparticles, the cyclohexane solutions with NaYF_4_:Er^3+^/Yb^3+^@NaYF_4_:Tm^3+^/Yb^3+^ core-shell particles and NaYF_4_:Er^3+^/Yb^3+^ nanoparticles were again used, and 980 nm fiber laser was adopted as excitation source. The procedure for photothermal conversion measurements is designed as follows. The solution containing nanoparticles is continuously irradiated by 980 nm laser, and then the UC spectra are measured at different time when the laser works at the current of 2.0 A. For the sake of studying the influence of core-shell particles’ content in solution on the heat generation, three solutions containing 5 Wt% naked NaYF_4_:Er^3+^/Yb^3+^ nanoparticles (named as solution I), 6 Wt% (solution II) and 13 Wt% (solution III) NaYF_4_:Er^3+^/Yb^3+^@NaYF_4_:Tm^3+^/Yb^3+^ core-shell particles in 7 mL cyclohxane were prepared, and 2 mL of each solution was used for the spectral measurements.

**Figure 5:**
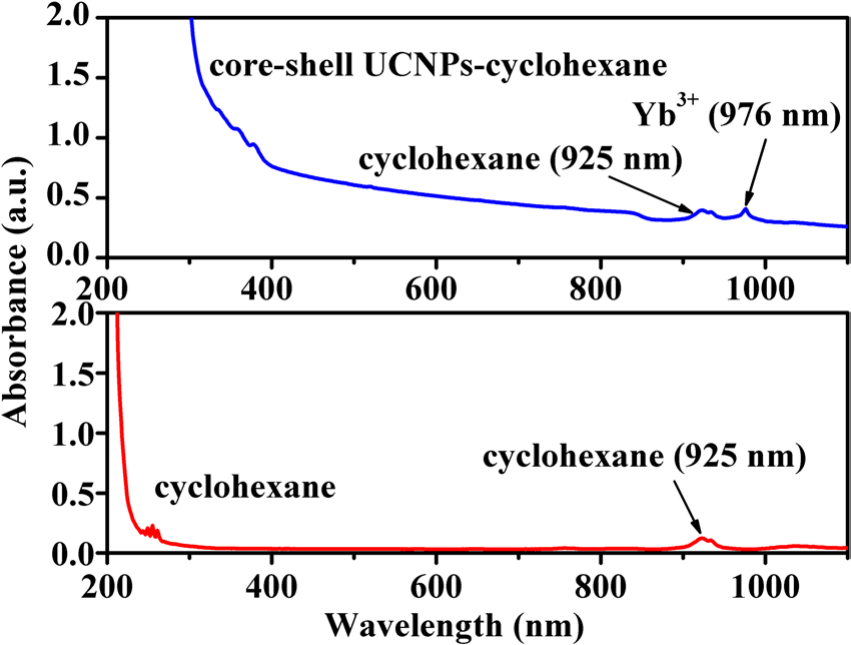
Absorption spectra of pure cyclohexane (control) and solution III.

To confirm that the heat generation of the system was caused by the nanoparticles but not cyclohexane via absorbing 980 nm irradiation, the absorption spectra of the pure cyclohexane and solution III were measured and are shown in Fig. 5. The absorption peak at about 925 nm can be seen in both solutions, which originates from the solvent cyclohexane. It should be noted that the absorption of pure cyclohexane at 980 nm is very weak, and the absorbed light may convert to other lights with different wavelengths or transform to heat energy. To examine the emissions of pure cyclohexane under 980 nm excitation, its emission spectra in the ranges from 200–900 nm on F-4600 (Hitachi) and from 900–2250 nm on NIRQuest-256 (Ocean Optics) were measured, and no any emissions were observed, thus implying that the absorbed light energy by cyclohexane can completely converted into heat energy. This means that the heat generation by cyclohexane can not be neglected. The absorption peak at about 976 nm corresponding to the ^2^F_7/2_ → ^2^F_5/2_ transition of Yb^3+^ is observed in solution III. The intense absorption centered at 976 nm in solution III containing core-shell nanoparticles is prerequisite for effective photothermal conversion, but this does not mean that the photothermal conversion can be achieved. Therefore, the photothermal conversion behavior should be further experimentally checked.

**Figure 6:**
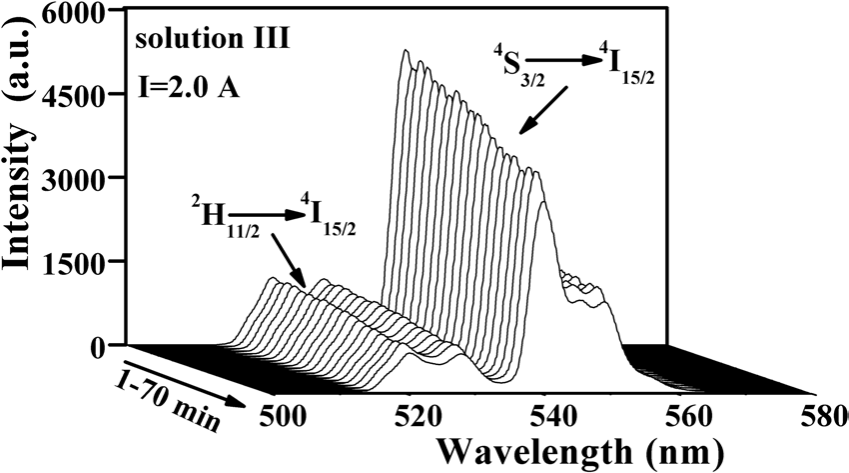
UC emission spectra of core-shell particles functioned as nano-calorifiers in solution III excited by 980 nm laser working at 2 A for 70 min.

**Figure 7:**
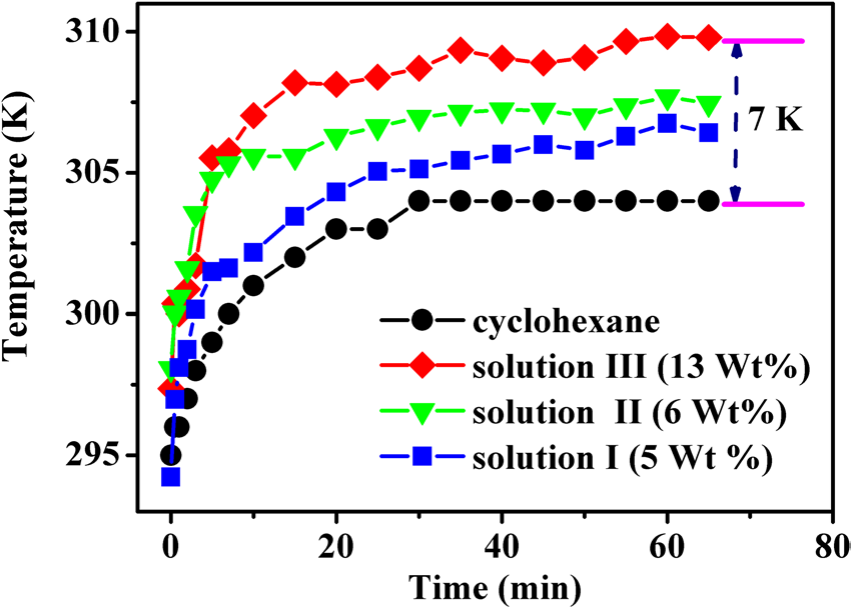
Relationship between the temperature of cyclohexane, solutions (I, II and III) and the laser irradiation time. Solid dots show the experimental data, solid curves indicate the variation trend.

As an example, Fig. 6 shows the UC spectra for solution III measured at different moments within 70 min under irradiation of 980 nm laser working at current of 2.0 A. To find the change of solution temperature with time, the UC emission intensity ratios ***R*** (***I***_***H***_ ***/I***_***S***_) were calculated, and then the solution temperatures at different moments were derived based on the obtained temperature response curve (Fig. 4a). Hereby, the relations between the temperature of all the solutions and laser irradiation time are shown in Fig. 7. For comparison, the time-dependent temperature for the pure cyclohexane was also given in Fig. 7. The temperature for the pure cyclohexane was measured with a thermocouple under the same experimental condition. From Fig. 7 it can be seen that the temperature of pure cyclohexane grows steadily with the increase of irradiation time within 30 min, and then keeps unchanged after reaching around 304 K, thus indicating that a thermal equilibrium is achieved in the studied system. This means that though the absorption of pure cyclohexane is weak at 980 nm, the photothermal conversion can not be ignored. This observed result for pure cyclohexane coincides with the deduction we made based on Fig. 5. In fact, the absorption of body fluid is also existent in the practical applications of PTT. Therefore, the investigation for photothermal conversion in solution environment may have more practical significance than that in solid powders. It can be found from Fig. 7 that the other solutions with nanoparticles display a similar variation trend of temperature toward irradiation time as the pure cyclohexane does, but they are really different from each others. The temperature increasing rate for solution I at initial moments is larger than that of pure cyclohexane, and the temperature at the final thermal equilibrium is higher than that of cyclohexane. These results indicate that NaYF_4_:Er^3+^/Yb^3+^ nanoparticles have contribution to the photothermal conversion. In comparison with solution I, the solution II and III exhibit more effective photothermal conversion since the final thermal equilibrium temperatures are higher, and the temperature increasing rates in initial stage of laser irradiation are larger. It should also be noted that solution III, which contains more amounts of core-shell particles than that of solution II, displays more effective photothermal conversion behavior. Additionally, though solution I and solution II contain approximately equal amount of nanoparticles (naked NaYF_4_:Er^3+^/Yb^3+^ core in solution I; NaYF_4_:Er^3+^/Yb^3+^@NaYF_4_:Tm^3+^/Yb^3+^ core-shell in solution II), the final equilibrium temperature of solution II is much higher than that of solution I, thus indicating that the NaYF_4_:Tm^3+^/Yb^3+^ shell plays important role in the photothermal conversion. It should be emphasized that a net temperature incensement of about 15 K from room temperature 295 K to final equilibrium temperature around 310 K can be obtained in solution III. The net temperature increment is around 7 K (see Fig. 7) when the contribution of heat generation by the solvent (cyclohexane) was deducted. It should be mentioned that less than one fifth of volume for the solution but not the full volume were irradiated by the laser beam. Meanwhile, it should also be noted that this 7 K increment is accomplished in the solution with low amount of the studied core-shell nanoparticles (photothermal agent), and the solution in a thermally open environment, thus it can be expected that a 6 K temperature increment from 37 (normal body temperature) to 43 °C (effective tumor treatment temperature) can be obtained by increasing amount of the core-shell nanoparticles deposited on tumor tissues which are surrounded by other tissues other than the open environment, since the temperature increment is proportional to the absorbed heat quality.

## Conclusions

The core-shell structured NaYF_4_:Er^3+^/Yb^3+^@NaYF_4_:Tm^3+^/Yb^3+^ nanoparticles were successfully synthesized via a thermolysis reaction, and characterized by means of XRD, SEM, TEM/HRTEM, from which it was found that the average core diameter was 19.2 nm and shell thickness was about ﻿2.0﻿ nm. In assistance of water bath heating the optical temperature sensing for the NaYF_4_:Er^3+^/Yb^3+^@NaYF_4_:Tm^3+^/Yb^3+^ core-shell nanoparticles was studied and compared with naked NaYF_4_:Er^3+^/Yb^3+^ core nanoparticles. It was found that the coating of photothermal converting NaYF_4_:Tm^3+^/Yb^3+^ shell did not obviously injure the temperature sensing performance of the core-shell particles, but improved the luminescence intensities of the thermally coupled ^2^H_11/2_ and ^4^S_3/2_ levels, that further made the optical signal measurements easy. The photothermal conversion behavior for the core-shell nanoparticles were studied, and it was found that the cyclohexane solution with NaYF_4_:Er^3+^/Yb^3+^@NaYF_4_:Tm^3+^/Yb^3+^ core-shell nanoparticles exhibited more effective photothermal conversion than that of the NaYF_4_:Er^3+^/Yb^3+^ nanoparticles, and a net temperature increment of 7 K was achieved.

## Experimental section

### Materials

Yttrium oxide (Y_2_O_3_, 99.99%), ytterbium oxide (Yb_2_O_3_, 99.99%), erbium oxide (Er_2_O_3_, 99.99%) and thulium oxide (Tm_2_O_3_, 99.99%) were purchased from Shanghai Second Chemical Reagent Factory (China). Other chemicals including cyclohexane, absolute ethanol, methanol, sodium hydroxide and ammonium fluoride were analytical grade reagents which were purchased from Tianjin Reagent Chemicals Co., Ltd (China). Oleic acid (OA, 90%) and 1-octadecene (ODE, 90%) were purchased from Sigma-Aldrich and Aladdin, respectively.

### Synthesis of lanthanide chloride with crystal water

A certain amount of RE_2_O_3_ (RE = Y, Er, Yb, Tm), water and hydrochloric acid (the volume ratio of hydrochloric acid to water is 1: 1) were added into a 250 mL beaker, then the mixture was heated under vigorous stirring until all the oxides were dissolved. Next, the transparent solution was re-crystallized five times with adding distilled water repeatedly. Lastly, the RECl_3_·6H_2_O (RE = Y, Er, Yb, Tm) samples were obtained when their corresponding crystallized products were well dried in air at 80°C for 12 h.

### Synthesis of NaYF_4_: Er^3+^/Yb^3+^ core UCNPs

NaYF_4_: Er^3+^/Yb^3+^ core particles were synthesized by a thermal decomposition route. As an example, a typical procedure is presented below. A total amount of 2 mmol RECl_3_·6H_2_O (RE:78 mol% Y^3+^, 20 mol% Yb^3+^ and 2 mol% Er^3+^), 12 mL OA and 30 ml ODE were added into a 100 ml three-necked flask which was then vacuumized for 30 min to remove oxygen under magnetic stirring at room temperature. Next, the flask containing the mixture was heated to 150 °C to dissolve lanthanide chloride, and a yellow transparent solution under vacuum with magnetic stirring was formed. When the solution cooled down to room temperature naturally, 10 mL methanol solution with 5 mmol NaOH and 8 mmol NH_4_F was dropped into the flask slowly. After stirring and reacting at room temperature, the mixed solution was then heated to 100 °C in N_2_ atmosphere and kept for 1 h to remove residual methanol, oxygen and water. Subsequently, the solution was heated to 310 °C, vigorously stirred and hold at this temperature for 1 h in N_2_ atmosphere. After cooling down to room temperature, the final products were collected with absolute ethanol and centrifugated at 9000 rpm for 10 min. The white precipitate was washed three times with absolute ethanol/cyclohexane (3:1 v/v). Finally, the NPs were dispersed in 5 mL of cyclohexane for further characterizations.

### Synthesis of NaYF_4_: Er/Yb@NaYF_4_: Tm/Yb core-shell structure

Following the same synthesis route above, the shell precursor solution containing 2 mmol RE_2_O_3_·6H_2_O (RE:79.5% mol Y^3+^, 20%mol Yb^3+^, 0.5%mol Tm^3+^), 12 mL OA and 30 ml ODE were prepared in the reaction vessel, after cooling down to room temperature, 2 mmol core product was added into the reaction vessel before adding methanol solution. After that the temperature was again increased up to 100 °C in N_2_ atmosphere and kept for 1 h to remove cyclohexane. After removing cyclohexane, the following synthesis procedure is the same as that of core particles presented above.

### Characterization

The crystalline structure of the samples was characterized by XRD (X-ray diffractometer using Cu-Kα1 radiation source, λ = 0.15406 nm, SHIMADZU, Japan). The XRD data in 2θ ranging from 10° to 80° were collected with a scanning step size of 0.02°. Morphologies and microscopic structures were observed by a field emission SEM (FE-SEM, SUPRA 55 SAPPHIRE, RIGAKU, Janpan) and a high resolution TEM (HR-TEM, JEM-2100F, JOEL, Japan). The UC emission spectra were recorded by using F-4600 spectrophotometer (HITACHI, Japan) under excitation of an externally introduced 980 nm fiber laser. It should be mentioned that if without specific statement, the excitation power density was around 0.24 × 10^2^ and 1.29 × 10^2^ W/cm^2^ when the working currents of 980 nm laser were 1.0 and 2.0 A, respectively. The reason why the low power was used for the temperature calibration experiments is to avoid the laser-irradiation-induced heating effect. The temperature controlling experiments were carried out with quartz cuvette (10 ^m^/_m_) heated by water bath, and a thermocouple was used to monitor the solution temperature.
